# GloBody Technology: Detecting Anti-Drug Antibody against VH/VL domains

**DOI:** 10.1038/s41598-020-58041-3

**Published:** 2020-02-05

**Authors:** Gauri K. Saxena, Ioannis Theocharopoulos, Nisha Thaslima Aziz, Meleri Jones, Sharmilee Gnanapavan, Gavin Giovannoni, Klaus Schmierer, James A. Garnett, David Baker, Angray S. Kang

**Affiliations:** 1 BartsMS, Blizard Institute, Barts and the London School of Medicine and Dentistry, Queen Mary University of London, London, E1 2AT United Kingdom; 2 School of Biological and Chemical Sciences, Queen Mary University of London, Mile End Road, London, E1 4NS United Kingdom; 3 Blizard Institute, Barts and the London School of Medicine and Dentistry, Queen Mary University of London, London, E1 2AT United Kingdom; 4 Clinical Board: Medicine (Neuroscience), The Royal London Hospital, Barts Health NHS Trust, London, E1 1BB United Kingdom; 5 Centre for Host-Microbiome Interactions, Kings College London, London, United Kingdom; 6 Centre for Oral Immunobiology and Regenerative Medicine, Dental Institute, Barts and the London School of Medicine and Dentistry, Queen Mary University of London, London, E1 2AT United Kingdom

**Keywords:** Biomarkers, Neurology

## Abstract

The occurrence of anti-drug antibodies following administration of therapeutic monoclonal antibody to patients is a growing problem that is attracting attention from frontline clinicians. Ideally, an initial indicative point of care test would provide guidance to seek testing approved by the regulatory authorities. Here we describe a platform for the detection of IgG anti-drug antibodies that may provide an initial screen for all therapeutic monoclonal antibodies. Synthetic genes encoding Nanoluciferase polypeptides were inserted between the variable heavy and light domain encoding region of known antibody drugs (alemtuzumab and adalimumab) to generate recombinant single chain GloBodies, which retain the drug antibody paratopes and Nanoluciferase activity. In the presence of anti-drug antibodies, the GloBody is bound by specific IgG in the sample. These complexes are captured on immobilised Protein G and the luciferase activity determined. The amount of light generated being indicative of the anti-drug IgG antibody levels in serum. It should be possible to assemble GloBody reagents for all therapeutic monoclonal antibodies and adapt the capture phase to include additional specific isotypes. The assay has the potential to be developed for use with a drop of blood allowing initial pre-screening in a point of care setting.

## Introduction

The immunogenicity of therapeutic monoclonal antibodies (mAbs) is a major concern for patient safety and for biopharmaceutical companies developing products for unmet needs. The anti-drug antibody (ADA) response influences pharmacokinetic, pharmacodynamic and clinical efficacy of the antibody drug. Since immunogenicity in humans is difficult to predict, ADA assay development, with guidance from the regulatory authorities, Food and Drugs Administration (FDA) and the European Medicines Agency (EMA) is part of the preclinical drug development pathway. Post-marketing monitoring of ADA provides guidance to physicians with respect to patient safety and well-being and to the drug sponsor/developers a measure of the mAbs potential immunogenicity issues that may not have arisen during clinical trials. With newly approved therapies, ADA assays are not always readily available for independent assessment. The current ADA detection platforms include solid phase extraction with acid dissociation^1^, affinity capture elution^2^, precipitation and dissociation^3^, antigen binding test and bridging immunoassays either in solution or on a solid phase, which is the most commonly used format (reviewed extensively elsewhere)^4–6^. These approaches have some limitations. Briefly, in the bridging assays, the capture and detection reagents require optimisation on a case-by-case basis for capture and detection (i.e., for immobilisation, biotin/enzyme/fluorophore labelling). This has partially been resolved by fusing Nanoluciferase (Nluc) to the C-terminus of the heavy chains of the drug antibody to generate a uniform IgG based reporter^7^. As with all bridging assays, the assumption is that one antibody-binding site of the ADA binds to the capture component and the other is free to interact with the detection drug, whereas both sites binding to either the capture or detection reagent is a possibility and thus theoretically underestimate ADA levels. All the above require individual drug reagent, a standard ADA and assay optimisation, dedicated specialised laboratory equipment and are ideal for central testing facilities. With mAbs dominating the biologics therapeutic space in a wide range of diseases and conditions, our objective was to develop a flexible ADA assay platform that can be adopted in a non-specialist setting. In this report we describe an approach that may complement the assays used in the drug development program (pre-clinical and clinical) and for post-marketing vigilance with the potential for point of care use for biologic drug ADA IgG screening.

As an exemplar biologic, we investigated Campath-1H, the first humanised monoclonal antibody^8^ also known as alemtuzumab (Lemtrada) that is used to treat relapsing multiple sclerosis (RMS). In 2014, the marketing authorisation for alemtuzumab recommended a dosage of 12 mg/day administered by intravenous infusion for two treatment courses. The initial treatment course lasts five consecutive days, followed a year later by the second treatment course of three consecutive days. In multiple sclerosis (MS), alemtuzumab induces ADA in about 85% of patients following the second treatment cycle^9,10^. Recently a third and potentially a fourth round of treatment in RMS with alemtuzumab was approved by the EMA^11^. A test for IgG ADA following the second treatment course prior to the third and subsequent rounds of treatment is needed as potentially neutralizing ADA may have developed^10^. In this communication, we describe a novel IgG ADA assay for the detection of IgG anti-alemtuzumab binding antibody in people with MS treated with this drug. The assay has the potential to be developed for use with a drop of blood allowing initial pre-screening in a point of care setting and identifying individuals for testing for neutralising ADA using a cell based assay.

## Results

### GloBody design

**Figure 1:**
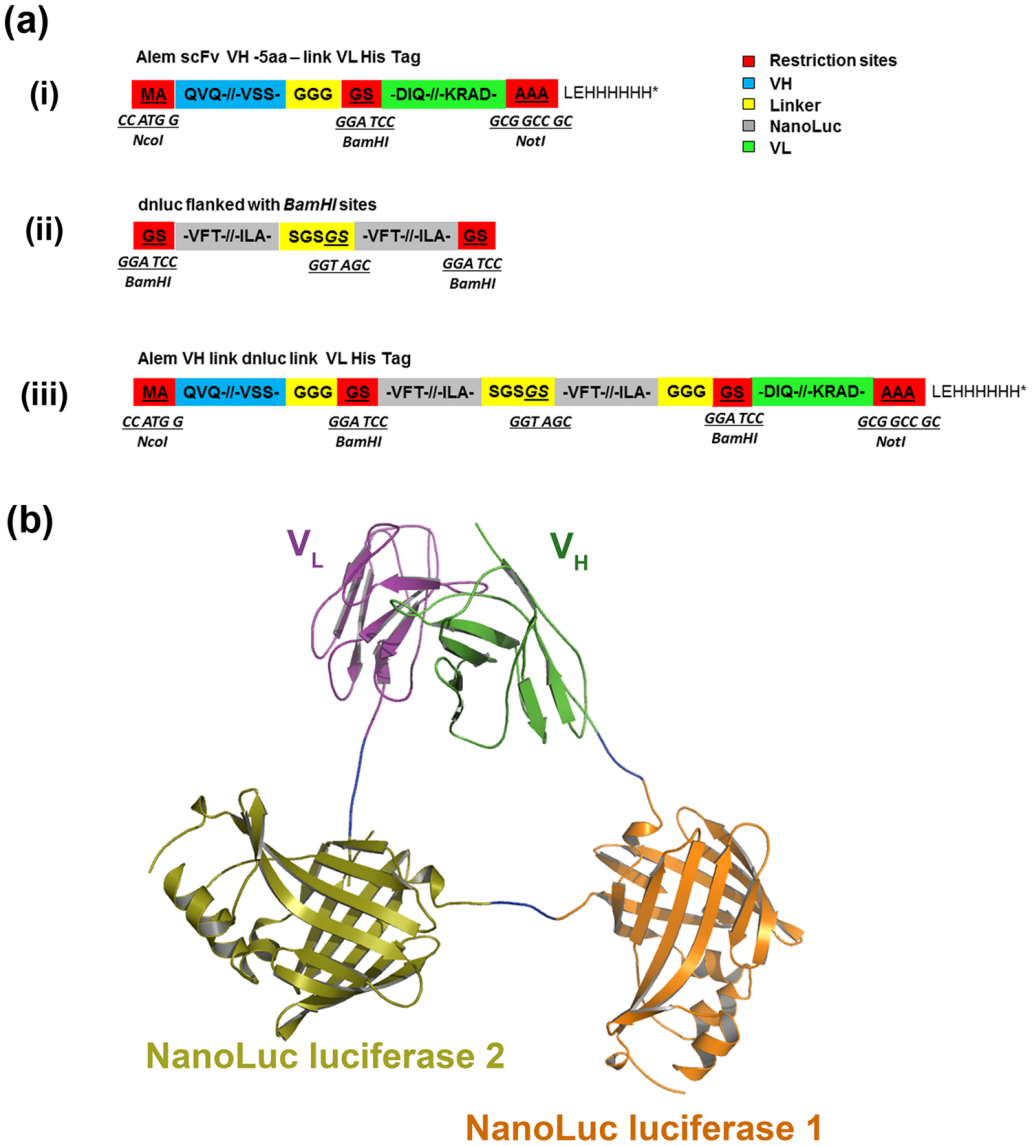
(**a**) A schematic for the assembly of a GloBody. (i) The Alemtuzumab scFv was designed with *NcoI*/*NotI* directional cloning sites and a 5 amino acid linker between the VH and VL with a unique in frame *BamHI* site. (ii) A dual Nanoluc was designed flanked by in-frame *BamHI* sites and inserted in to the scFv to generate (iii) GloBody expression cassette. (**b**) A 3D model of the Alemtuzumab/ tandem dual Nanoluc luciferase fusion antibody. Molecular model (ribbon representation) of the CAMPATH-1H antigen-binding fragment (Fv) (PDB 1BEY) fused with the two Nanoluc luciferase (PDB 5IBO) separated by a short linker sequence. The Fv variable region heavy chain (VH) is coloured green whilst the Fv variable region light chain (VL) is coloured purple. First Nanoluc (orange) and the second nanoluc (olive) is fused in between these domains and is separated by two short amino acid linker sequences (blue).

Since the ADA with the potential to be inhibitory are directed against the variable regions of the antibody drug, a minimal structure for detecting ADA would require the VH and VL assembled to form a functional binding site mimicking the structure of the antibody drug. Previously, we described antibody variable domains linked with monomeric fluorescent proteins. The docking of monomeric fluorescent protein in-between the variable regions VH/VL of a single chain antibody (scFv) of known antibodies resulted in VH/VL interface interactions to create soluble fluorescent antibodies each with a single binding site and a single fluorophore^12,13^. Here we replaced the monomeric fluorescent domain with enzymes capable of retaining the VH/VL interface and generating an amplifiable signal. The enzyme selected was an ATP independent luciferase isolated from a deep-sea shrimp (*Oplophorus gracilirostris*) that had undergone extensive *in vitro* directed evolution to generate a stable robust 19 kDa Nluc^14^. A 38.75 kDa dual tandem Nluc (dnluc) designed assembled and inserted between the VH/VL of alemtuzumab scFv to generate Alem GloBody as shown in Fig. 1. The GloBody lack of immunoglobulin constant regions precludes interaction with protein G (or anti-Fc capture antibody). Additionally, GloBody based on adalimumab VH/VL domains was also prepared.

### GloBody assay

**Figure 2:**
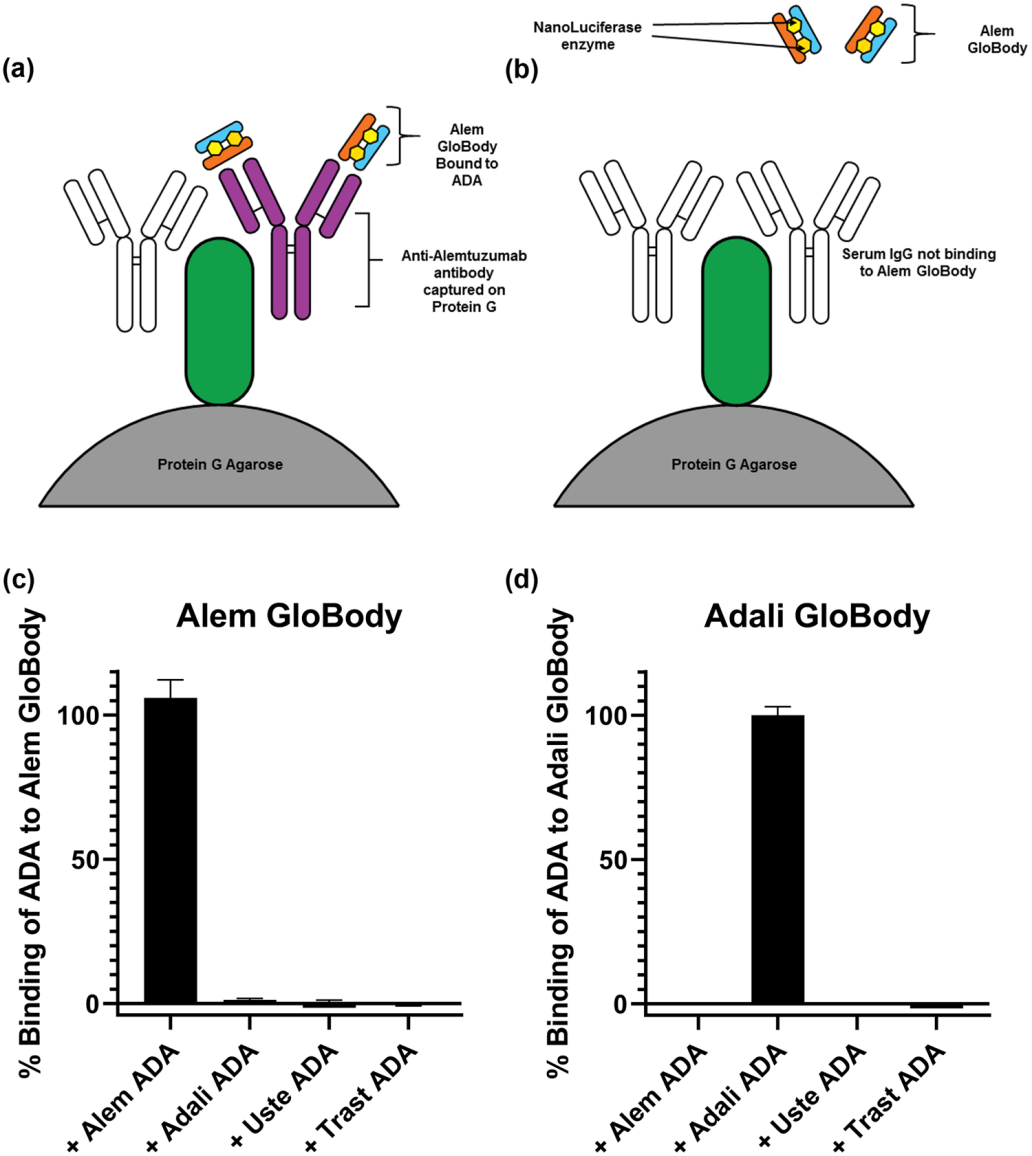
(**a**) A schematic of the assay format. Anti-alemtuzumab antibodies present in serum bind to the Alem Globody. The total IgG is captured on Protein G allowing detection of the retained luciferase. In panel (**b**) in the absence of ADA the Globody is not retained. To determine the specificity of the assay, (**c**) Alem GloBody was incubated with ADA against alemtuzumab, adalimumab, ustekinumab and trastuzumab. Luciferase signal was only detected with ADA against alemtuzumab. (**d**) Conversely, Adali GloBody was incubated with ADA against alemtuzumab, adalimumab, ustekinumab and trastuzumab. Luciferase signal was detected with ADA against adalimumab, but no luciferase signal was detected with ADA against alemtuzumab, ustekinumab or trastuzumab.

In the assay, acidification of serum dissociates pre-existing ADA-drug complexes, addition of a vast excess of Alem GloBody (with neutralising solution) allows the ADA to bind to the GloBody in solution. Capture of the IgG in the sample using Protein G retains the ADA bound Alem GloBody as depicted in Fig. 2(a,b). After washing to remove the excess unbound GloBody reagent, the retained enzyme activity is determined with the light generated being proportional to the amount of ADA in the sample.

The specificity of the GloBodies based on alemtuzumab and adalimumab variable regions were determined in a binding assay using commercially available human monoclonal anti-drug antibodies spiked into human control sera. The Alem GloBody had the highest binding to the anti-alemtuzumab antibody with negligible binding to ADA against other drugs (Fig. 2c). Likewise Adali GloBody had highest binding to its corresponding ADA, but not the other ADA’s (Fig. 2d). Additional ADA’s against ustekinumab and trastuzumab did not bind to either of the GloBodies tested (Fig. 2c,d).

### GloBody assay with patient samples

**Figure 3:**
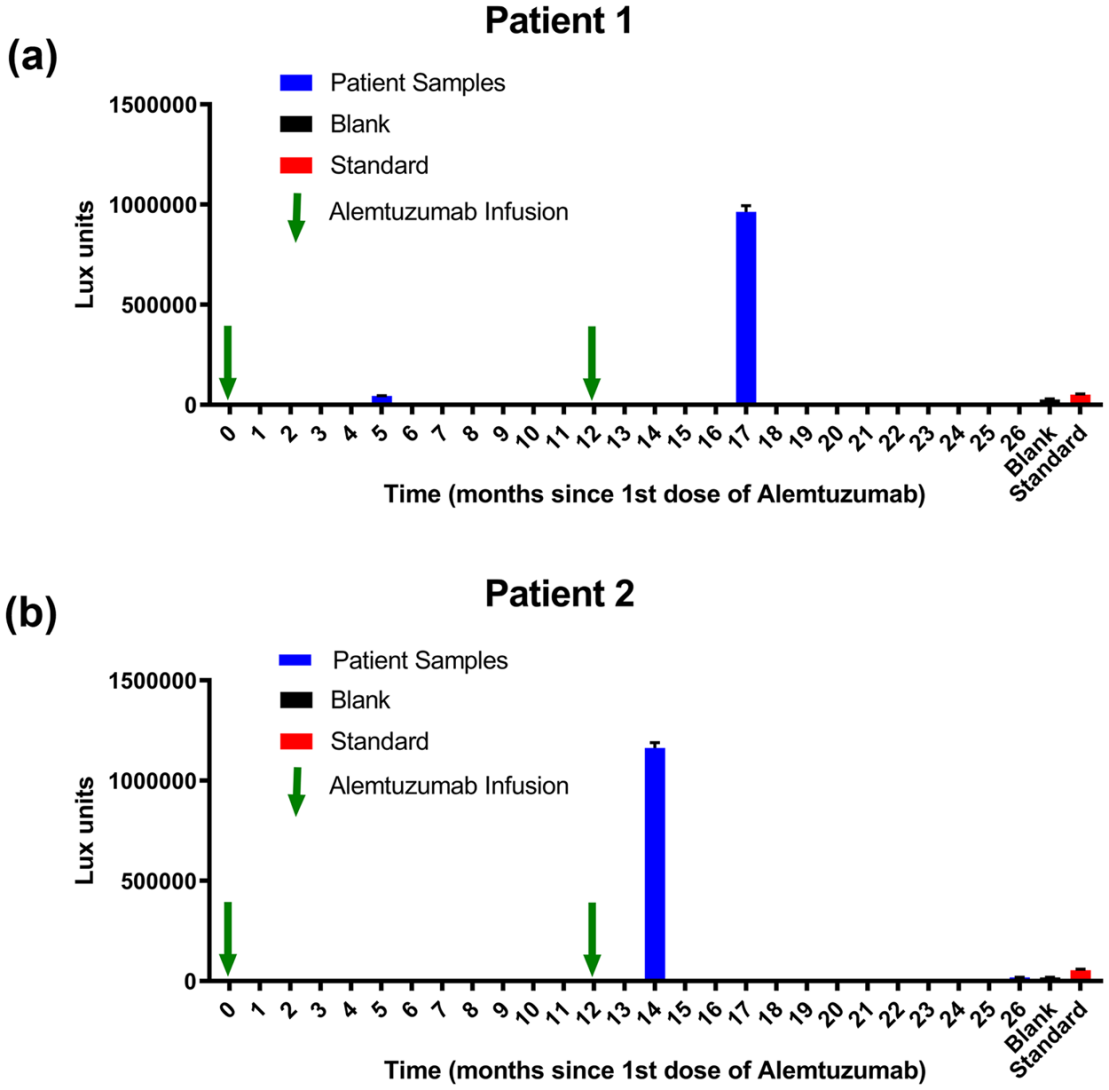
Detection of ADA in serum samples from patients treated with alemtuzumab. (**a**) In patient 1, initially dosed with alemtuzumab at month 0 and a 1^st^ serum sample taken at month 5, then dosed at month 12 and a 2^nd^ serum sample taken at month 17. The control blank and the 50 µg/mL ADA positive included as reference points. Luciferase activity above the limit of detection were observed at both time points. In patient 2, (**b**) dosed with alemtuzumab at month 0 and then at month 12 prior to the 1^st^ serum sample at month 14 and a 2nd serum sample taken at month 26. The control blank and the 50 µg/mL ADA positive included as reference points. Luciferase activity above the limit of detection was observed at 2 months after the 2^nd^ infusion which then decreased to below the LoD at month 26.

In this proof of concept study, we identified patients that had seroconverted to making IgG anti-alemtuzumab antibodies following treatment with alemtuzumab. Unlike conventional immunoassays with a single analyte, the ADA response is polyclonal, a mix of antibodies with a range of specificities and affinities each at different concentrations, thus a true standard is unattainable since each individuals response to the therapeutic antibody will be different. In the absence of ADA standards, a qualitative readout, with reference to the limit of blank (LoB)^15^ may be used. The capture of the GloBody is dependent on ADA in the sample and a signal statistically higher than the blank is positive. In this case, the LoB is the highest luminescence value expected to be found when replicates of a blank sample containing no ADA are tested. In this study the value is defined by LoB = mean luminescence_blank_ + 2.58(standard deviation_blank_), values greater than this would suggest the presence of ADA with a confidence of 99%. The LoB calculated using blank serum in the analysis of patients 1 was 3.186 × 10^4^ lux units suggesting the presence of ADA at 5 months (4.46 × 10^4^ lux units) following the first administration of the drug and a rise (9.63 × 10^5^ lux units) after the second round of treatment as shown in Fig. 3(a). With patient 2 the LoB was 2.061 × 10^4^ lux units and the first serum sample collected at month 14 in March 2016 and then again a year later (month 26). In both cases, we see a significant rise in ADA following the 2^nd^ administration of the drug whether assayed after 2 or 5 months. In the case of patient 2 the sample taken 14 months after the second round of treatment, the ADA had declined to below the LoB (1.85 × 10^4^ lux units) as shown in Fig. 3(b). In the absence of an ideal standard these criteria could be applied generally to all ADA assays carried out using the GloBody platform.

Drug tolerance is not addressed in this study since as stated on the Lemtrada package insert^16^ there is a rapid disappearance of alemtuzumab from the systemic circulation, becoming undetectable by 1 month post-treatment in all patients. The elimination half-life was approximately 2 weeks and was comparable between courses. The serum concentrations were generally undetectable (<60 ng/mL) within approximately 30 days following each treatment course. The samples provided for this retrospective study were obtained 2, 5 and 14 months after the drug was infused. However, for other antibody therapies administered more frequently with a longer half-life, the tolerance issue will need to be addressed on a case-by-case basis.

In the case of alemtuzumab, the interval between infusion and ADA testing is critical. With the availability of a test, that requires ~20 µL of serum (i.e., a drop of blood) it will be possible to frequently monitor ADA to determine when levels are rising or falling and likely to lead to drug failure or success.

## Conclusions

Since the GloBody design is modular; it is applicable to any therapeutic mAb and CAR-T cell therapy for monitoring ADA against the VH/VL combinations. For multimeric, i.e., bispecific or trispecific each VH/VL combinantion may be assayed, providing additional information on whether the ADA is directed to one or more specificities. The current format can be used without requiring further manipulation (i.e., conjugation of biotin, Alexa dyes or coupling/immobilisation of a capture drug to the biosensor chips) since each binding site has exactly 2 Nluc enzymes covalently attached between the VH and VL domains. The capture of immunoglobulins with Protein G, as demonstrated here or potentially with anti-Fc specific immobilised antibody is the basis of a general assay platform. The capture step for immunoglobulin allows for isotypes specificity if required. The GloBody reagents for all therapeutic mAbs offer a platform for developing assays for monitoring the development of ADA’s and is not reliant on a bridging format or the availability of a standard. This assay detects binding antibodies and warrants a larger cohort study with detailed analysis to correlate ADA levels with drug efficacy.

## Methods

Structures of Campath-1H^17^, Adalimumab and Nluc were downloaded from Protein Data Bank (PDB) database (PBD: 1BEY, 4NYL and 5IBO respectively). The Alem GloBody model was then constructed through manual localization of the individual monomers and manual building of inter-domain linkers using COOT^18^ followed by energy minimisation in CNS^19^ as shown in Fig. 1. Plasmid pK10, previously constructed in our laboratory based on pET-26b vector (Novagen)^13^ was modified by replacing the T7 promoter with a LacZ promoter. All primers purchased from Merck. The amino acid sequence of the variable domains of alemtuzumab and adalimumab were accessed from DrugBank via accession numbers DB00087and DB00051 respectively and scFv assembled with the antibody variable domains in VH-VL orientation. Synthetic DNA sequences of Alemtuzumab and Adalimumab antibody scFv’s and a synthetic Nluc optimised for *Escherichia coli* codon usage and purchased (Genscript). Plasmid pNluc.1 encoding Nluc obtained from Promega. The expression vectors assembled using a combination of conventional restriction digest/ligation, overlap extension PCR and Gibson assembly^20^. Gibson assembly was used to construct the plasmids pK10 Alem GloBody and pK10 Adali GloBody.

NEB 5α and NEB Express I^q^ *E. coli* strains (NEB) were used for the cloning and for recombinant protein production respectively. During cloning *E. coli* cells were grown on LB agar plates with kanamycin (50 μg/mL). Plasmid DNA was isolated using GenElute Plasmid Miniprep Kit (Merck) and DNA from the gel purified using Gel Extraction Kit (NEB). The chemical competent *E. coli* cells were transformed using standard heat shock methods following the instructions provided. Restriction and modification enzymes purchased from New England Biolabs (NEB). Plasmid constructs confirmed by DNA sequence analysis. All PCR amplifications carried out using Q5 polymerase (NEB)

The *scFv* insert directionally cloned into the pK10 vector as *NcoI/NotI* fragment. The competent *E.coli* NEB 5α cells transformed with the assembly mixtures and the clones selected on the LB plates containing kanamycin. Positive clones confirmed by DNA sequencing. The *nluc* derived from pNluc.1 and synNluc respectively were PCR amplified and joined together with a Ser Gly Ser Gly Ser encoding linker to make dnluc chimeras in VH-dnluc-VL orientation. Bacterial cultures assayed for Nluc activity and selected clones confirmed by plasmid DNA sequencing. All sequences and construct details provided in the supplementary information.

To produce Alem GloBody, *E. coli* NEB Express I^q^ (NEB) cells were transformed with the plasmid and plated onto LB agar supplemented with kanamycin sulphate (50 μg/mL). A single colony NEB Express I^q^ transformed with the plasmid added to 200 mL Overnight Express Auto-Induction media^21^ with 100 µg/mL kanamycin, grown with shaking at 275 rpm for 24 hr. A 50 mL aliquots of culture pelleted at 877 × g for 20 min, the supernatant discarded and each pellet re-suspended in a 10 mL of lysis solution containing (1 mL Bugbuster × 10, 8 mL PBS, 1 mL lysozyme (10 mg/mL) and 1 µL benzonase (20 units) and placed on a rolling platform for 1 h. The lysate transferred into 2 mL Eppendorf tubes and centrifuged at 10,000 × g at 4 °C for another 20 min. The resulting supernatant collected and filtered through a 0.45 µm filter. A 3 mL HisPur Co-NTA (Thermo Fisher) column equilibrated at room temperature and the storage buffer drained. Tris 50 mM (2 × 6 mL) containing 10 mM Imidazole added to the column and drained. The filtered lysate pooled and imidazole added to give a final concentration of 10 mM loaded onto the column and allowed to drain by gravity. The column was washed twice with a 7.5 mL solution of Tris 50 mM, 0.5 M NaCl, 10 mM Imidazole. The bound protein eluted with a 31 mL solution of Tris 50 mM, 0.5 M NaCl, 500 mM Imidazole. Approximately 1.4 mL fractions were collected (E1-E22) and assayed for luciferase activity. E4-E9, the highest luciferase activity fractions were pooled, diluted in PBS with 50% glycerol and aliquot (~8.4 × 10^7^ lux units) stored at −80 °C until required (each vial had a total volume of 30 µL). GloBody based on the variable regions of Adalimumab and was also assembled and prepared as described above.

### Assay specificity

Human monoclonal anti-alemtuzumab IgG (Bio-Rad HCA-199 Affinity KD, 0.2 nM), anti-adalimumab IgG (Bio-Rad HCA-204 Affinity KD, 0.06 nM), anti-ustekinumab IgG (Bio-Rad HCA-210 Affinity KD, 0.2 nM) and anti-trastuzumab IgG (Bio-Rad HCA-177 Affinity KD, 0.02 nM) stocks 0.5 mg/mL) were diluted in human control sera~ final concentration 50 µg/mL. To 20 µL of each of the diluted ADA either Alemtuzumab, Adalimumab or Ustekinumab GloBody was added (~6.5 × 106 lux activity units) in 1 mL PBST. A 50% mixture of Protein G agarose equilibrated in PBS (60 µL, capacity ~ 600 µg IgG) added and incubated at ambient temperature for 2 h on rotating wheel. The agarose pelleted 6,200 × g for 1 min and the supernatant carefully aspirated leaving behind the protein G agarose and residual buffer (~0.1 mL) 1.3 mL PBST added to wash the resin. The wash process repeated six times to remove unbound Alem GloBody. The agarose resin transferred to Micro-Spin Columns (Pierce) and washed twice with PBS 350 µL with centrifugation at 6,200 × g. To elute the IgG and retained Alem GloBody 50 µL 0.1 M Glycine pH2.7 was added to the agarose at ambient temperature for 5 min and centrifuged at 16,200 × g for 1 min into collection tube containing 6 µL 0.1 M Tris pH9.0. Neutralized eluate (15 µL) was added to 0.1 mL NanoGlo substrate (Promega) (20 µL furimazine in 1 mL Glo Buffer) in triplicate and after 10 min luminescence measured on a CLARIOstar plus plate reader using the pre-set nanoluciferase setting and the data plotted using GraphPad Prism

### Anti-Drug-Antibody assay format

The BartsHealth NHS informed consent form was in accordance with the guidelines of the Declaration of Helsinki. The samples were taken as part of routine care of MS patients with excess being stored for research. Blood from consented MS patients treated with alemtuzumab were collected and processed between March 2016 and 2017 and the serum stored at −20 °C until required. Human control sera (Merck, H4522) used as a base line control. A human monoclonal anti-alemtuzumab IgG (Bio-Rad HCA-199 Affinity K_D_, 0.2 nM, stock 0.5 mg/mL) diluted in human control sera~ final concentration 50 µg/mL used as a single point positive. To dissociate preformed ADA-drug complexes, to 20 µL of serum (assuming a range of ~ 10–15 µg/ µL max 300 µg IgG) sample acidified with the addition of 50 µl of 0.1 M glycine pH2.7. Alem GloBody ~6.5 × 10^6^ lux activity units 0.1 mL in PBST was mixed with 6 µL 0.1 M Tris pH 9.0 then the whole added to the acidified serum sample and the volume made up to 1 mL with PBST and placed on rotating wheel for 30 min at ambient temperature. A 50% mixture of Protein G agarose equilibrated in PBS (60 µL, capacity ~ 600 µg IgG) added and the procedure described above followed. Serum test samples, standard (50 µg/mL ADA spiked into blank serum) and blank serum were assayed.

## Supplementary information

Suplemental Information.
