# Supplementary material for: GloBody Technology: Detecting Anti-Drug Antibody against VH/VL domains
Source: Sci Rep. 2020 Feb 5;10:1860. doi: 10.1038/s41598-020-58041-3 (PMC7002611; doi:10.1038/s41598-020-58041-3)
Supplement: Supplementary file 1 — Suplemental Information. [file 41598_2020_58041_MOESM1_ESM.docx]

Title: GloBody Technology: Detecting Anti-Drug Antibody against VH/VL domains

Authors: Gauri K Saxena, Ioannis Theocharopoulos, Nisha Thaslima Aziz, Meleri Jones,

Sharmilee Gnanapavan, Gavin Giovannoni, Klaus Schmierer, James A Garnett, David Baker and Angray S Kang

**Suplemental Sequences**

Dnluc sequence RED Promega nluc BLUE Syn nluc BamHI sites underlined

GGATCCGTCTTCACACTCGAAGATTTCGTTGGGGACTGGCGACAGACAGCCGGCTACAACCTGGACCAAGTCCTTGAACAGGGAGGTGTGTCCAGTTTGTTTCAGAATCTCGGGGTGTCCGTAACTCCGATCCAAAGGATTGTCCTGAGCGGTGAAAATGGGCTGAAGATCGACATCCATGTCATCATCCCGTATGAAGGTCTGAGCGGCGACCAAATGGGCCAGATCGAAAAAATTTTTAAGGTGGTGTACCCTGTGGATGATCATCACTTTAAGGTGATCCTGCACTATGGCACACTGGTAATCGACGGGGTTACGCCGAACATGATCGACTATTTCGGACGGCCGTATGAAGGCATCGCCGTGTTCGACGGCAAAAAGATCACTGTAACAGGGACCCTGTGGAACGGCAACAAAATTATCGACGAGCGCCTGATCAACCCCGACGGCTCCCTGCTGTTCCGAGTAACCATCAACGGAGTGACCGGCTGGCGGCTGTGCGAACGCATTCTGGCGTCTGGTTCAGGAAGTGTGTTTACCCTGGAAGATTTTGTGGGCGATTGGCGCCAGACCGCGGGCTATAACCTGGATCAGGTGCTGGAACAGGGCGGCGTGAGCAGCCTGTTTCAGAACCTGGGCGTGAGCGTGACCCCGATTCAGCGCATTGTGCTGAGCGGCGAAAACGGCCTGAAAATTGATATTCATGTGATTATTCCGTATGAAGGCCTGAGCGGCGATCAGATGGGCCAGATTGAAAAAATTTTTAAAGTGGTGTATCCGGTGGATGATCATCATTTTAAAGTGATTCTGCATTATGGCACCCTGGTGATTGATGGCGTGACCCCGAACATGATTGATTATTTTGGCCGCCCGTATGAAGGCATTGCGGTGTTTGATGGCAAAAAAATTACCGTGACCGGCACCCTGTGGAACGGCAACAAAATTATTGATGAACGCCTGATTAACCCGGATGGCAGCCTGCTGTTTCGCGTGACCATTAACGGCGTGACCGGCTGGCGCCTGTGTGAGCGTATACTTGCAGGCGGCGGCGGATCC

Translated

GSVFTLEDFVGDWRQTAGYNLDQVLEQGGVSSLFQNLGVSVTPIQRIVLSGENGLKIDIHVIIPYEGLSGDQMGQIEKIFKVVYPVDDHHFKVILHYGTLVIDGVTPNMIDYFGRPYEGIAVFDGKKITVTGTLWNGNKIIDERLINPDGSLLFRVTINGVTGWRLCERILASGSGSVFTLEDFVGDWRQTAGYNLDQVLEQGGVSSLFQNLGVSVTPIQRIVLSGENGLKIDIHVIIPYEGLSGDQMGQIEKIFKVVYPVDDHHFKVILHYGTLVIDGVTPNMIDYFGRPYEGIAVFDGKKITVTGTLWNGNKIIDERLINPDGSLLFRVTINGVTGWRLCERILAGGGGS

Alem GloBody NcoI, BamHI and NotI sites underlined

CCATGGCGCAGGTGCAGCTGCAGGAAAGCGGCCCGGGCCTGGTGCGCCCGAGCCAGACCCTGAGCCTGACCTGCACCGTGAGCGGCTTTACCTTTACCGATTTTTATATGAACTGGGTGCGCCAGCCGCCGGGCCGCGGCCTGGAATGGATTGGCTTTATTCGCGATAAAGCGAAAGGCTATACCACCGAATATAACCCGAGCGTGAAAGGCCGCGTGACCATGCTGGTGGATACCAGCAAAAACCAGTTTAGCCTGCGCCTGAGCAGCGTGACCGCGGCGGATACCGCGGTGTATTATTGCGCGCGCGAAGGCCATACCGCGGCGCCGTTTGATTATTGGGGCCAGGGCAGCCTGGTGACCGTGAGCAGCGGTGGCGGGGGATCCGTCTTCACACTCGAAGATTTCGTTGGGGACTGGCGACAGACAGCCGGCTACAACCTGGACCAAGTCCTTGAACAGGGAGGTGTGTCCAGTTTGTTTCAGAATCTCGGGGTGTCCGTAACTCCGATCCAAAGGATTGTCCTGAGCGGTGAAAATGGGCTGAAGATCGACATCCATGTCATCATCCCGTATGAAGGTCTGAGCGGCGACCAAATGGGCCAGATCGAAAAAATTTTTAAGGTGGTGTACCCTGTGGATGATCATCACTTTAAGGTGATCCTGCACTATGGCACACTGGTAATCGACGGGGTTACGCCGAACATGATCGACTATTTCGGACGGCCGTATGAAGGCATCGCCGTGTTCGACGGCAAAAAGATCACTGTAACAGGGACCCTGTGGAACGGCAACAAAATTATCGACGAGCGCCTGATCAACCCCGACGGCTCCCTGCTGTTCCGAGTAACCATCAACGGAGTGACCGGCTGGCGGCTGTGCGAACGCATTCTGGCGTCTGGTTCAGGAAGTGTGTTTACCCTGGAAGATTTTGTGGGCGATTGGCGCCAGACCGCGGGCTATAACCTGGATCAGGTGCTGGAACAGGGCGGCGTGAGCAGCCTGTTTCAGAACCTGGGCGTGAGCGTGACCCCGATTCAGCGCATTGTGCTGAGCGGCGAAAACGGCCTGAAAATTGATATTCATGTGATTATTCCGTATGAAGGCCTGAGCGGCGATCAGATGGGCCAGATTGAAAAAATTTTTAAAGTGGTGTATCCGGTGGATGATCATCATTTTAAAGTGATTCTGCATTATGGCACCCTGGTGATTGATGGCGTGACCCCGAACATGATTGATTATTTTGGCCGCCCGTATGAAGGCATTGCGGTGTTTGATGGCAAAAAAATTACCGTGACCGGCACCCTGTGGAACGGCAACAAAATTATTGATGAACGCCTGATTAACCCGGATGGCAGCCTGCTGTTTCGCGTGACCATTAACGGCGTGACCGGCTGGCGCCTGTGTGAGCGTATACTTGCAGGCGGCGGCGGATCCGATATTCAGATGACCCAGAGCCCGAGCAGCCTGAGCGCGAGCGTGGGCGATCGCGTGACCATTACCTGCAAAGCGAGCCAGAACATTGATAAATATCTGAACTGGTATCAGCAGAAACCGGGCAAAGCGCCGAAACTGCTGATTTATAACACCAACAACCTGCAGACCGGCGTGCCGAGCCGCTTTAGCGGCAGCGGCAGCGGCACCGATTTTACCTTTACCATTAGCAGCCTGCAGCCGGAAGATATTGCGACCTATTATTGCCTGCAGCATATTAGCCGCCCGCGCACCTTTGGCCAGGGCACCAAAGTGGAAATTAAACGCGCGGATGCGGCCGC

Translated

MAQVQLQESGPGLVRPSQTLSLTCTVSGFTFTDFYMNWVRQPPGRGLEWIGFIRDKAKGYTTEYNPSVKGRVTMLVDTSKNQFSLRLSSVTAADTAVYYCAREGHTAAPFDYWGQGSLVTVSSGGGGSVFTLEDFVGDWRQTAGYNLDQVLEQGGVSSLFQNLGVSVTPIQRIVLSGENGLKIDIHVIIPYEGLSGDQMGQIEKIFKVVYPVDDHHFKVILHYGTLVIDGVTPNMIDYFGRPYEGIAVFDGKKITVTGTLWNGNKIIDERLINPDGSLLFRVTINGVTGWRLCERILASGSGSVFTLEDFVGDWRQTAGYNLDQVLEQGGVSSLFQNLGVSVTPIQRIVLSGENGLKIDIHVIIPYEGLSGDQMGQIEKIFKVVYPVDDHHFKVILHYGTLVIDGVTPNMIDYFGRPYEGIAVFDGKKITVTGTLWNGNKIIDERLINPDGSLLFRVTINGVTGWRLCERILAGGGGSDIQMTQSPSSLSASVGDRVTITCKASQNIDKYLNWYQQKPGKAPKLLIYNTNNLQTGVPSRFSGSGSGTDFTFTISSLQPEDIATYYCLQHISRPRTFGQGTKVEIKRADAA

VH nLuc syn nLuc VL

pK10 ALEM VH-dnluc-VL-His (aka pK10 Alem GloBody)

AGATCTAACGCAATTAATGTGAGTTAGCTCACTCATTAGGCACCCCAGGCTTTACACTTTATGCTTCCGGCTCGTATGTTGTGTGGAATTGTGAGCGGATAACAATTTCCCTCTAGAAATAATTTTGTTTAACTTTAAGAAGGAGATATACAT**ATG**AAATACCTGCTGCCGACCGCTGCTGCTGGTCTGCTGCTCCTCGCTGCCCAGCCGGCCATGGCGCAGGTGCAGCTGCAGGAAAGCGGCCCGGGCCTGGTGCGCCCGAGCCAGACCCTGAGCCTGACCTGCACCGTGAGCGGCTTTACCTTTACCGATTTTTATATGAACTGGGTGCGCCAGCCGCCGGGCCGCGGCCTGGAATGGATTGGCTTTATTCGCGATAAAGCGAAAGGCTATACCACCGAATATAACCCGAGCGTGAAAGGCCGCGTGACCATGCTGGTGGATACCAGCAAAAACCAGTTTAGCCTGCGCCTGAGCAGCGTGACCGCGGCGGATACCGCGGTGTATTATTGCGCGCGCGAAGGCCATACCGCGGCGCCGTTTGATTATTGGGGCCAGGGCAGCCTGGTGACCGTGAGCAGCGGTGGCGGG**GGATCC**GTCTTCACACTCGAAGATTTCGTTGGGGACTGGCGACAGACAGCCGGCTACAACCTGGACCAAGTCCTTGAACAGGGAGGTGTGTCCAGTTTGTTTCAGAATCTCGGGGTGTCCGTAACTCCGATCCAAAGGATTGTCCTGAGCGGTGAAAATGGGCTGAAGATCGACATCCATGTCATCATCCCGTATGAAGGTCTGAGCGGCGACCAAATGGGCCAGATCGAAAAAATTTTTAAGGTGGTGTACCCTGTGGATGATCATCACTTTAAGGTGATCCTGCACTATGGCACACTGGTAATCGACGGGGTTACGCCGAACATGATCGACTATTTCGGACGGCCGTATGAAGGCATCGCCGTGTTCGACGGCAAAAAGATCACTGTAACAGGGACCCTGTGGAACGGCAACAAAATTATCGACGAGCGCCTGATCAACCCCGACGGCTCCCTGCTGTTCCGAGTAACCATCAACGGAGTGACCGGCTGGCGGCTGTGCGAACGCATTCTGGCGTCTGGTTCAGGAAGTGTGTTTACCCTGGAAGATTTTGTGGGCGATTGGCGCCAGACCGCGGGCTATAACCTGGATCAGGTGCTGGAACAGGGCGGCGTGAGCAGCCTGTTTCAGAACCTGGGCGTGAGCGTGACCCCGATTCAGCGCATTGTGCTGAGCGGCGAAAACGGCCTGAAAATTGATATTCATGTGATTATTCCGTATGAAGGCCTGAGCGGCGATCAGATGGGCCAGATTGAAAAAATTTTTAAAGTGGTGTATCCGGTGGATGATCATCATTTTAAAGTGATTCTGCATTATGGCACCCTGGTGATTGATGGCGTGACCCCGAACATGATTGATTATTTTGGCCGCCCGTATGAAGGCATTGCGGTGTTTGATGGCAAAAAAATTACCGTGACCGGCACCCTGTGGAACGGCAACAAAATTATTGATGAACGCCTGATTAACCCGGATGGCAGCCTGCTGTTTCGCGTGACCATTAACGGCGTGACCGGCTGGCGCCTGTGTGAGCGTATACTTGCAGGCGGCGGC**GGATCC**GATATTCAGATGACCCAGAGCCCGAGCAGCCTGAGCGCGAGCGTGGGCGATCGCGTGACCATTACCTGCAAAGCGAGCCAGAACATTGATAAATATCTGAACTGGTATCAGCAGAAACCGGGCAAAGCGCCGAAACTGCTGATTTATAACACCAACAACCTGCAGACCGGCGTGCCGAGCCGCTTTAGCGGCAGCGGCAGCGGCACCGATTTTACCTTTACCATTAGCAGCCTGCAGCCGGAAGATATTGCGACCTATTATTGCCTGCAGCATATTAGCCGCCCGCGCACCTTTGGCCAGGGCACCAAAGTGGAAATTAAACGCGCGGAT**GCGGCCGC**ACTCGAGCATCATCATCACCATCACTAAAGTGATGGTGATGATGATGTGCAGCCGGATCAAGCTTGATCCGGCTGCTAACAAAGCCCGAAAGGAAGCTGAGTTGGCTGCTGCCACCGCTGAGCAATAACTAGCATAACCCCTTGGGGCCTCTAAACGGGTCTTGAGGGGTTTTTTGCTGAAAGGAGGAACTATATCCGGATTGGCGAATGGGACGCGCCCTGTAGCGGCGCATTAAGCGCGGCGGGTGTGGTGGTTACGCGCAGCGTGACCGCTACACTTGCCAGCGCCCTAGCGCCCGCTCCTTTCGCTTTCTTCCCTTCCTTTCTCGCCACGTTCGCCGGCTTTCCCCGTCAAGCTCTAAATCGGGGGCTCCCTTTAGGGTTCCGATTTAGTGCTTTACGGCACCTCGACCCCAAAAAACTTGATTAGGGTGATGGTTCACGTAGTGGGCCATCGCCCTGATAGACGGTTTTTCGCCCTTTGACGTTGGAGTCCACGTTCTTTAATAGTGGACTCTTGTTCCAAACTGGAACAACACTCAACCCTATCTCGGTCTATTCTTTTGATTTATAAGGGATTTTGCCGATTTCGGCCTATTGGTTAAAAAATGAGCTGATTTAACAAAAATTTAACGCGAATTTTAACAAAATATTAACGCTTACAATTTAGGTGGCACTTTTCGGGGAAATGTGCGCGGAACCCCTATTTGTTTATTTTTCTAAATACATTCAAATATGTATCCGCTCATGAATTAATTCTTAGAAAAACTCATCGAGCATCAAATGAAACTGCAATTTATTCATATCAGGATTATCAATACCATATTTTTGAAAAAGCCGTTTCTGTAATGAAGGAGAAAACTCACCGAGGCAGTTCCATAGGATGGCAAGATCCTGGTATCGGTCTGCGATTCCGACTCGTCCAACATCAATACAACCTATTAATTTCCCCTCGTCAAAAATAAGGTTATCAAGTGAGAAATCACCATGAGTGACGACTGAATCCGGTGAGAATGGCAAAAGTTTATGCATTTCTTTCCAGACTTGTTCAACAGGCCAGCCATTACGCTCGTCATCAAAATCACTCGCATCAACCAAACCGTTATTCATTCGTGATTGCGCCTGAGCGAGACGAAATACGCGATCGCTGTTAAAAGGACAATTACAAACAGGAATCGAATGCAACCGGCGCAGGAACACTGCCAGCGCATCAACAATATTTTCACCTGAATCAGGATATTCTTCTAATACCTGGAATGCTGTTTTCCCGGGGATCGCAGTGGTGAGTAACCATGCATCATCAGGAGTACGGATAAAATGCTTGATGGTCGGAAGAGGCATAAATTCCGTCAGCCAGTTTAGTCTGACCATCTCATCTGTAACATCATTGGCAACGCTACCTTTGCCATGTTTCAGAAACAACTCTGGCGCATCGGGCTTCCCATACAATCGATAGATTGTCGCACCTGATTGCCCGACATTATCGCGAGCCCATTTATACCCATATAAATCAGCATCCATGTTGGAATTTAATCGCGGCCTAGAGCAAGACGTTTCCCGTTGAATATGGCTCATAACACCCCTTGTATTACTGTTTATGTAAGCAGACAGTTTTATTGTTCATGACCAAAATCCCTTAACGTGAGTTTTCGTTCCACTGAGCGTCAGACCCCGTAGAAAAGATCAAAGGATCTTCTTGAGATCCTTTTTTTCTGCGCGTAATCTGCTGCTTGCAAACAAAAAAACCACCGCTACCAGCGGTGGTTTGTTTGCCGGATCAAGAGCTACCAACTCTTTTTCCGAAGGTAACTGGCTTCAGCAGAGCGCAGATACCAAATACTGTCCTTCTAGTGTAGCCGTAGTTAGGCCACCACTTCAAGAACTCTGTAGCACCGCCTACATACCTCGCTCTGCTAATCCTGTTACCAGTGGCTGCTGCCAGTGGCGATAAGTCGTGTCTTACCGGGTTGGACTCAAGACGATAGTTACCGGATAAGGCGCAGCGGTCGGGCTGAACGGGGGGTTCGTGCACACAGCCCAGCTTGGAGCGAACGACCTACACCGAACTGAGATACCTACAGCGTGAGCTATGAGAAAGCGCCACGCTTCCCGAAGGGAGAAAGGCGGACAGGTATCCGGTAAGCGGCAGGGTCGGAACAGGAGAGCGCACGAGGGAGCTTCCAGGGGGAAACGCCTGGTATCTTTATAGTCCTGTCGGGTTTCGCCACCTCTGACTTGAGCGTCGATTTTTGTGATGCTCGTCAGGGGGGCGGAGCCTATGGAAAAACGCCAGCAACGCGGCCTTTTTACGGTTCCTGGCCTTTTGCTGGCCTTTTGCTCACATGTTCTTTCCTGCGTTATCCCCTGATTCTGTGGATAACCGTATTACCGCCTTTGAGTGAGCTGATACCGCTCGCCGCAGCCGAACGACCGAGCGCAGCGAGTCAGTGAGCGAGGAAGCGGAAGAGCGCCTGATGCGGTATTTTCTCCTTACGCATCTGTGCGGTATTTCACACCGCAATGGTGCACTCTCAGTACAATCTGCTCTGATGCCGCATAGTTAAGCCAGTATACACTCCGCTATCGCTACGTGACTGGGTCATGGCTGCGCCCCGACACCCGCCAACACCCGCTGACGCGCCCTGACGGGCTTGTCTGCTCCCGGCATCCGCTTACAGACAAGCTGTGACCGTCTCCGGGAGCTGCATGTGTCAGAGGTTTTCACCGTCATCACCGAAACGCGCGAGGCAGCTGCGGTAAAGCTCATCAGCGTGGTCGTGAAGCGATTCACAGATGTCTGCCTGTTCATCCGCGTCCAGCTCGTTGAGTTTCTCCAGAAGCGTTAATGTCTGGCTTCTGATAAAGCGGGCCATGTTAAGGGCGGTTTTTTCCTGTTTGGTCACTGATGCCTCCGTGTAAGGGGGATTTCTGTTCATGGGGGTAATGATACCGATGAAACGAGAGAGGATGCTCACGATACGGGTTACTGATGATGAACATGCCCGGTTACTGGAACGTTGTGAGGGTAAACAACTGGCGGTATGGATGCGGCGGGACCAGAGAAAAATCACTCAGGGTCAATGCCAGCGCTTCGTTAATACAGATGTAGGTGTTCCACAGGGTAGCCAGCAGCATCCTGCGATGCAGATCCGGAACATAATGGTGCAGGGCGCTGACTTCCGCGTTTCCAGACTTTACGAAACACGGAAACCGAAGACCATTCATGTTGTTGCTCAGGTCGCAGACGTTTTGCAGCAGCAGTCGCTTCACGTTCGCTCGCGTATCGGTGATTCATTCTGCTAACCAGTAAGGCAACCCCGCCAGCCTAGCCGGGTCCTCAACGACAGGAGCACGATCATGCGCACCCGTGGGGCCGCCATGCCGGCGATAATGGCCTGCTTCTCGCCGAAACGTTTGGTGGCGGGACCAGTGACGAAGGCTTGAGCGAGGGCGTGCAAGATTCCGAATACCGCAAGCGACAGGCCGATCATCGTCGCGCTCCAGCGAAAGCGGTCCTCGCCGAAAATGACCCAGAGCGCTGCCGGCACCTGTCCTACGAGTTGCATGATAAAGAAGACAGTCATAAGTGCGGCGACGATAGTCATGCCCCGCGCCCACCGGAAGGAGCTGACTGGGTTGAAGGCTCTCAAGGGCATCGGTCGAGATCCCGGTGCCTAATGAGTGAGCTAACTTACATTAATTGCGTTGCGCTCACTGCCCGCTTTCCAGTCGGGAAACCTGTCGTGCCAGCTGCATTAATGAATCGGCCAACGCGCGGGGAGAGGCGGTTTGCGTATTGGGCGCCAGGGTGGTTTTTCTTTTCACCAGTGAGACGGGCAACAGCTGATTGCCCTTCACCGCCTGGCCCTGAGAGAGTTGCAGCAAGCGGTCCACGCTGGTTTGCCCCAGCAGGCGAAAATCCTGTTTGATGGTGGTTAACGGCGGGATATAACATGAGCTGTCTTCGGTATCGTCGTATCCCACTACCGAGATATCCGCACCAACGCGCAGCCCGGACTCGGTAATGGCGCGCATTGCGCCCAGCGCCATCTGATCGTTGGCAACCAGCATCGCAGTGGGAACGATGCCCTCATTCAGCATTTGCATGGTTTGTTGAAAACCGGACATGGCACTCCAGTCGCCTTCCCGTTCCGCTATCGGCTGAATTTGATTGCGAGTGAGATATTTATGCCAGCCAGCCAGACGCAGACGCGCCGAGACAGAACTTAATGGGCCCGCTAACAGCGCGATTTGCTGGTGACCCAATGCGACCAGATGCTCCACGCCCAGTCGCGTACCGTCTTCATGGGAGAAAATAATACTGTTGATGGGTGTCTGGTCAGAGACATCAAGAAATAACGCCGGAACATTAGTGCAGGCAGCTTCCACAGCAATGGCATCCTGGTCATCCAGCGGATAGTTAATGATCAGCCCACTGACGCGTTGCGCGAGAAGATTGTGCACCGCCGCTTTACAGGCTTCGACGCCGCTTCGTTCTACCATCGACACCACCACGCTGGCACCCAGTTGATCGGCGCGAGATTTAATCGCCGCGACAATTTGCGACGGCGCGTGCAGGGCCAGACTGGAGGTGGCAACGCCAATCAGCAACGACTGTTTGCCCGCCAGTTGTTGTGCCACGCGGTTGGGAATGTAATTCAGCTCCGCCATCGCCGCTTCCACTTTTTCCCGCGTTTTCGCAGAAACGTGGCTGGCCTGGTTCACCACGCGGGAAACGGTCTGATAAGAGACACCGGCATACTCTGCGACATCGTATAACGTTACTGGTTTCACATTCACCACCCTGAATTGACTCTCTTCCGGGCGCTATCATGCCATACCGCGAAAGGTTTTGCGCCATTCGATGGTGTCCGGGATCTCGACGCTCTCCCTTATGCGACTCCTGCATTAGGAAGCAGCCCAGTAGTAGGTTGAGGCCGTTGAGCACCGCCGCCGCAAGGAATGGTGCATGCAAGGAGATGGCGCCCAACAGTCCCCCGGCCACGGGGCCTGCCACCATACCCACGCCGAAACAAGCGCTCATGAGCCCGAAGTGGCGAGCCCGATCTTCCCCATCGGTGATGTCGGCGATATAGGCGCCAGCAACCGCACCTGTGGCGCCGGTGATGCCGGCCACGATGCGTCCGGCGTAGAGGATCG

Translated

MKYLLPTAAAGLLLLAAQPAMAQVQLQESGPGLVRPSQTLSLTCTVSGFTFTDFYMNWVRQPPGRGLEWIGFIRDKAKGYTTEYNPSVKGRVTMLVDTSKNQFSLRLSSVTAADTAVYYCAREGHTAAPFDYWGQGSLVTVSSGGGGSVFTLEDFVGDWRQTAGYNLDQVLEQGGVSSLFQNLGVSVTPIQRIVLSGENGLKIDIHVIIPYEGLSGDQMGQIEKIFKVVYPVDDHHFKVILHYGTLVIDGVTPNMIDYFGRPYEGIAVFDGKKITVTGTLWNGNKIIDERLINPDGSLLFRVTINGVTGWRLCERILASGSGSVFTLEDFVGDWRQTAGYNLDQVLEQGGVSSLFQNLGVSVTPIQRIVLSGENGLKIDIHVIIPYEGLSGDQMGQIEKIFKVVYPVDDHHFKVILHYGTLVIDGVTPNMIDYFGRPYEGIAVFDGKKITVTGTLWNGNKIIDERLINPDGSLLFRVTINGVTGWRLCERILAGGGGSDIQMTQSPSSLSASVGDRVTITCKASQNIDKYLNWYQQKPGKAPKLLIYNTNNLQTGVPSRFSGSGSGTDFTFTISSLQPEDIATYYCLQHISRPRTFGQGTKVEIKRADAAALEHHHHHH*

PelB Leader VH Nluc Nluc VL His Tag

pK10 ADALI VH-dnluc-VL-His (aka pK10 Adali GloBody)

AGATCTAACGCAATTAATGTGAGTTAGCTCACTCATTAGGCACCCCAGGCTTTACACTTTATGCTTCCGGCTCGTATGTTGTGTGGAATTGTGAGCGGATAACAATTTCCCTCTAGAAATAATTTTGTTTAACTTTAAGAAGGAGATATACAT**ATG**AAATACCTGCTGCCGACCGCTGCTGCTGGTCTGCTGCTCCTCGCTGCCCAGCCGGCCATGGCGGAAGTGCAGCTGGTGGAAAGCGGCGGCGGCCTGGTGCAGCCGGGCCGCAGCCTGCGCCTGAGCTGCGCGGCGAGCGGCTTTACCTTTGATGATTATGCGATGCATTGGGTGCGCCAGGCGCCGGGCAAAGGCCTGGAATGGGTGAGCGCGATTACCTGGAACAGCGGCCATATTGATTATGCGGATAGCGTGGAAGGCCGCTTTACCATTAGCCGCGATAACGCGAAAAACAGCCTGTATCTGCAGATGAACAGCCTGCGCGCGGAAGATACCGCGGTGTATTATTGCGCGAAAGTGAGCTATCTGAGCACCGCGAGCAGCCTGGATTATTGGGGCCAGGGCACCCTGGTGACCGTGAGCAGCGCGAGCACC**GGATCC**GTCTTCACACTCGAAGATTTCGTTGGGGACTGGCGACAGACAGCCGGCTACAACCTGGACCAAGTCCTTGAACAGGGAGGTGTGTCCAGTTTGTTTCAGAATCTCGGGGTGTCCGTAACTCCGATCCAAAGGATTGTCCTGAGCGGTGAAAATGGGCTGAAGATCGACATCCATGTCATCATCCCGTATGAAGGTCTGAGCGGCGACCAAATGGGCCAGATCGAAAAAATTTTTAAGGTGGTGTACCCTGTGGATGATCATCACTTTAAGGTGATCCTGCACTATGGCACACTGGTAATCGACGGGGTTACGCCGAACATGATCGACTATTTCGGACGGCCGTATGAAGGCATCGCCGTGTTCGACGGCAAAAAGATCACTGTAACAGGGACCCTGTGGAACGGCAACAAAATTATCGACGAGCGCCTGATCAACCCCGACGGCTCCCTGCTGTTCCGAGTAACCATCAACGGAGTGACCGGCTGGCGGCTGTGCGAACGCATTCTGGCGTCTGGTTCAGGAAGTGTGTTTACCCTGGAAGATTTTGTGGGCGATTGGCGCCAGACCGCGGGCTATAACCTGGATCAGGTGCTGGAACAGGGCGGCGTGAGCAGCCTGTTTCAGAACCTGGGCGTGAGCGTGACCCCGATTCAGCGCATTGTGCTGAGCGGCGAAAACGGCCTGAAAATTGATATTCATGTGATTATTCCGTATGAAGGCCTGAGCGGCGATCAGATGGGCCAGATTGAAAAAATTTTTAAAGTGGTGTATCCGGTGGATGATCATCATTTTAAAGTGATTCTGCATTATGGCACCCTGGTGATTGATGGCGTGACCCCGAACATGATTGATTATTTTGGCCGCCCGTATGAAGGCATTGCGGTGTTTGATGGCAAAAAAATTACCGTGACCGGCACCCTGTGGAACGGCAACAAAATTATTGATGAACGCCTGATTAACCCGGATGGCAGCCTGCTGTTTCGCGTGACCATTAACGGCGTGACCGGCTGGCGCCTGTGTGAGCGTATACTTGCAGGCGGCGGC**GGATCC**GATATTCAGATGACCCAGAGCCCGAGCAGCCTGAGCGCGAGCGTGGGCGATCGCGTGACCATTACCTGCCGCGCGAGCCAGGGCATTCGCAACTATCTGGCGTGGTATCAGCAGAAACCGGGCAAAGCGCCGAAACTGCTGATTTATGCGGCGAGCACCCTGCAGAGCGGCGTGCCGAGCCGCTTTAGCGGCAGCGGCAGCGGCACCGATTTTACCCTGACCATTAGCAGCCTGCAGCCGGAAGATGTGGCGACCTATTATTGCCAGCGCTATAACCGCGCGCCGTATACCTTTGGCCAGGGCACCAAAGTGGAAATTAAACGCGCGGAT**GCGGCCGC**ACTCGAGCATCATCATCACCATCACTAAAGTGATGGTGATGATGATGTGCAGCCGGATCAAGCTTGATCCGGCTGCTAACAAAGCCCGAAAGGAAGCTGAGTTGGCTGCTGCCACCGCTGAGCAATAACTAGCATAACCCCTTGGGGCCTCTAAACGGGTCTTGAGGGGTTTTTTGCTGAAAGGAGGAACTATATCCGGATTGGCGAATGGGACGCGCCCTGTAGCGGCGCATTAAGCGCGGCGGGTGTGGTGGTTACGCGCAGCGTGACCGCTACACTTGCCAGCGCCCTAGCGCCCGCTCCTTTCGCTTTCTTCCCTTCCTTTCTCGCCACGTTCGCCGGCTTTCCCCGTCAAGCTCTAAATCGGGGGCTCCCTTTAGGGTTCCGATTTAGTGCTTTACGGCACCTCGACCCCAAAAAACTTGATTAGGGTGATGGTTCACGTAGTGGGCCATCGCCCTGATAGACGGTTTTTCGCCCTTTGACGTTGGAGTCCACGTTCTTTAATAGTGGACTCTTGTTCCAAACTGGAACAACACTCAACCCTATCTCGGTCTATTCTTTTGATTTATAAGGGATTTTGCCGATTTCGGCCTATTGGTTAAAAAATGAGCTGATTTAACAAAAATTTAACGCGAATTTTAACAAAATATTAACGCTTACAATTTAGGTGGCACTTTTCGGGGAAATGTGCGCGGAACCCCTATTTGTTTATTTTTCTAAATACATTCAAATATGTATCCGCTCATGAATTAATTCTTAGAAAAACTCATCGAGCATCAAATGAAACTGCAATTTATTCATATCAGGATTATCAATACCATATTTTTGAAAAAGCCGTTTCTGTAATGAAGGAGAAAACTCACCGAGGCAGTTCCATAGGATGGCAAGATCCTGGTATCGGTCTGCGATTCCGACTCGTCCAACATCAATACAACCTATTAATTTCCCCTCGTCAAAAATAAGGTTATCAAGTGAGAAATCACCATGAGTGACGACTGAATCCGGTGAGAATGGCAAAAGTTTATGCATTTCTTTCCAGACTTGTTCAACAGGCCAGCCATTACGCTCGTCATCAAAATCACTCGCATCAACCAAACCGTTATTCATTCGTGATTGCGCCTGAGCGAGACGAAATACGCGATCGCTGTTAAAAGGACAATTACAAACAGGAATCGAATGCAACCGGCGCAGGAACACTGCCAGCGCATCAACAATATTTTCACCTGAATCAGGATATTCTTCTAATACCTGGAATGCTGTTTTCCCGGGGATCGCAGTGGTGAGTAACCATGCATCATCAGGAGTACGGATAAAATGCTTGATGGTCGGAAGAGGCATAAATTCCGTCAGCCAGTTTAGTCTGACCATCTCATCTGTAACATCATTGGCAACGCTACCTTTGCCATGTTTCAGAAACAACTCTGGCGCATCGGGCTTCCCATACAATCGATAGATTGTCGCACCTGATTGCCCGACATTATCGCGAGCCCATTTATACCCATATAAATCAGCATCCATGTTGGAATTTAATCGCGGCCTAGAGCAAGACGTTTCCCGTTGAATATGGCTCATAACACCCCTTGTATTACTGTTTATGTAAGCAGACAGTTTTATTGTTCATGACCAAAATCCCTTAACGTGAGTTTTCGTTCCACTGAGCGTCAGACCCCGTAGAAAAGATCAAAGGATCTTCTTGAGATCCTTTTTTTCTGCGCGTAATCTGCTGCTTGCAAACAAAAAAACCACCGCTACCAGCGGTGGTTTGTTTGCCGGATCAAGAGCTACCAACTCTTTTTCCGAAGGTAACTGGCTTCAGCAGAGCGCAGATACCAAATACTGTCCTTCTAGTGTAGCCGTAGTTAGGCCACCACTTCAAGAACTCTGTAGCACCGCCTACATACCTCGCTCTGCTAATCCTGTTACCAGTGGCTGCTGCCAGTGGCGATAAGTCGTGTCTTACCGGGTTGGACTCAAGACGATAGTTACCGGATAAGGCGCAGCGGTCGGGCTGAACGGGGGGTTCGTGCACACAGCCCAGCTTGGAGCGAACGACCTACACCGAACTGAGATACCTACAGCGTGAGCTATGAGAAAGCGCCACGCTTCCCGAAGGGAGAAAGGCGGACAGGTATCCGGTAAGCGGCAGGGTCGGAACAGGAGAGCGCACGAGGGAGCTTCCAGGGGGAAACGCCTGGTATCTTTATAGTCCTGTCGGGTTTCGCCACCTCTGACTTGAGCGTCGATTTTTGTGATGCTCGTCAGGGGGGCGGAGCCTATGGAAAAACGCCAGCAACGCGGCCTTTTTACGGTTCCTGGCCTTTTGCTGGCCTTTTGCTCACATGTTCTTTCCTGCGTTATCCCCTGATTCTGTGGATAACCGTATTACCGCCTTTGAGTGAGCTGATACCGCTCGCCGCAGCCGAACGACCGAGCGCAGCGAGTCAGTGAGCGAGGAAGCGGAAGAGCGCCTGATGCGGTATTTTCTCCTTACGCATCTGTGCGGTATTTCACACCGCAATGGTGCACTCTCAGTACAATCTGCTCTGATGCCGCATAGTTAAGCCAGTATACACTCCGCTATCGCTACGTGACTGGGTCATGGCTGCGCCCCGACACCCGCCAACACCCGCTGACGCGCCCTGACGGGCTTGTCTGCTCCCGGCATCCGCTTACAGACAAGCTGTGACCGTCTCCGGGAGCTGCATGTGTCAGAGGTTTTCACCGTCATCACCGAAACGCGCGAGGCAGCTGCGGTAAAGCTCATCAGCGTGGTCGTGAAGCGATTCACAGATGTCTGCCTGTTCATCCGCGTCCAGCTCGTTGAGTTTCTCCAGAAGCGTTAATGTCTGGCTTCTGATAAAGCGGGCCATGTTAAGGGCGGTTTTTTCCTGTTTGGTCACTGATGCCTCCGTGTAAGGGGGATTTCTGTTCATGGGGGTAATGATACCGATGAAACGAGAGAGGATGCTCACGATACGGGTTACTGATGATGAACATGCCCGGTTACTGGAACGTTGTGAGGGTAAACAACTGGCGGTATGGATGCGGCGGGACCAGAGAAAAATCACTCAGGGTCAATGCCAGCGCTTCGTTAATACAGATGTAGGTGTTCCACAGGGTAGCCAGCAGCATCCTGCGATGCAGATCCGGAACATAATGGTGCAGGGCGCTGACTTCCGCGTTTCCAGACTTTACGAAACACGGAAACCGAAGACCATTCATGTTGTTGCTCAGGTCGCAGACGTTTTGCAGCAGCAGTCGCTTCACGTTCGCTCGCGTATCGGTGATTCATTCTGCTAACCAGTAAGGCAACCCCGCCAGCCTAGCCGGGTCCTCAACGACAGGAGCACGATCATGCGCACCCGTGGGGCCGCCATGCCGGCGATAATGGCCTGCTTCTCGCCGAAACGTTTGGTGGCGGGACCAGTGACGAAGGCTTGAGCGAGGGCGTGCAAGATTCCGAATACCGCAAGCGACAGGCCGATCATCGTCGCGCTCCAGCGAAAGCGGTCCTCGCCGAAAATGACCCAGAGCGCTGCCGGCACCTGTCCTACGAGTTGCATGATAAAGAAGACAGTCATAAGTGCGGCGACGATAGTCATGCCCCGCGCCCACCGGAAGGAGCTGACTGGGTTGAAGGCTCTCAAGGGCATCGGTCGAGATCCCGGTGCCTAATGAGTGAGCTAACTTACATTAATTGCGTTGCGCTCACTGCCCGCTTTCCAGTCGGGAAACCTGTCGTGCCAGCTGCATTAATGAATCGGCCAACGCGCGGGGAGAGGCGGTTTGCGTATTGGGCGCCAGGGTGGTTTTTCTTTTCACCAGTGAGACGGGCAACAGCTGATTGCCCTTCACCGCCTGGCCCTGAGAGAGTTGCAGCAAGCGGTCCACGCTGGTTTGCCCCAGCAGGCGAAAATCCTGTTTGATGGTGGTTAACGGCGGGATATAACATGAGCTGTCTTCGGTATCGTCGTATCCCACTACCGAGATATCCGCACCAACGCGCAGCCCGGACTCGGTAATGGCGCGCATTGCGCCCAGCGCCATCTGATCGTTGGCAACCAGCATCGCAGTGGGAACGATGCCCTCATTCAGCATTTGCATGGTTTGTTGAAAACCGGACATGGCACTCCAGTCGCCTTCCCGTTCCGCTATCGGCTGAATTTGATTGCGAGTGAGATATTTATGCCAGCCAGCCAGACGCAGACGCGCCGAGACAGAACTTAATGGGCCCGCTAACAGCGCGATTTGCTGGTGACCCAATGCGACCAGATGCTCCACGCCCAGTCGCGTACCGTCTTCATGGGAGAAAATAATACTGTTGATGGGTGTCTGGTCAGAGACATCAAGAAATAACGCCGGAACATTAGTGCAGGCAGCTTCCACAGCAATGGCATCCTGGTCATCCAGCGGATAGTTAATGATCAGCCCACTGACGCGTTGCGCGAGAAGATTGTGCACCGCCGCTTTACAGGCTTCGACGCCGCTTCGTTCTACCATCGACACCACCACGCTGGCACCCAGTTGATCGGCGCGAGATTTAATCGCCGCGACAATTTGCGACGGCGCGTGCAGGGCCAGACTGGAGGTGGCAACGCCAATCAGCAACGACTGTTTGCCCGCCAGTTGTTGTGCCACGCGGTTGGGAATGTAATTCAGCTCCGCCATCGCCGCTTCCACTTTTTCCCGCGTTTTCGCAGAAACGTGGCTGGCCTGGTTCACCACGCGGGAAACGGTCTGATAAGAGACACCGGCATACTCTGCGACATCGTATAACGTTACTGGTTTCACATTCACCACCCTGAATTGACTCTCTTCCGGGCGCTATCATGCCATACCGCGAAAGGTTTTGCGCCATTCGATGGTGTCCGGGATCTCGACGCTCTCCCTTATGCGACTCCTGCATTAGGAAGCAGCCCAGTAGTAGGTTGAGGCCGTTGAGCACCGCCGCCGCAAGGAATGGTGCATGCAAGGAGATGGCGCCCAACAGTCCCCCGGCCACGGGGCCTGCCACCATACCCACGCCGAAACAAGCGCTCATGAGCCCGAAGTGGCGAGCCCGATCTTCCCCATCGGTGATGTCGGCGATATAGGCGCCAGCAACCGCACCTGTGGCGCCGGTGATGCCGGCCACGATGCGTCCGGCGTAGAGGATCG

Translated

MKYLLPTAAAGLLLLAAQPAMAEVQLVESGGGLVQPGRSLRLSCAASGFTFDDYAMHWVRQAPGKGLEWVSAITWNSGHIDYADSVEGRFTISRDNAKNSLYLQMNSLRAEDTAVYYCAKVSYLSTASSLDYWGQGTLVTVSSASTGSVFTLEDFVGDWRQTAGYNLDQVLEQGGVSSLFQNLGVSVTPIQRIVLSGENGLKIDIHVIIPYEGLSGDQMGQIEKIFKVVYPVDDHHFKVILHYGTLVIDGVTPNMIDYFGRPYEGIAVFDGKKITVTGTLWNGNKIIDERLINPDGSLLFRVTINGVTGWRLCERILASGSGSVFTLEDFVGDWRQTAGYNLDQVLEQGGVSSLFQNLGVSVTPIQRIVLSGENGLKIDIHVIIPYEGLSGDQMGQIEKIFKVVYPVDDHHFKVILHYGTLVIDGVTPNMIDYFGRPYEGIAVFDGKKITVTGTLWNGNKIIDERLINPDGSLLFRVTINGVTGWRLCERILAGGGGSDIQMTQSPSSLSASVGDRVTITCRASQGIRNYLAWYQQKPGKAPKLLIYAASTLQSGVPSRFSGSGSGTDFTLTISSLQPEDVATYYCQRYNRAPYTFGQGTKVEIKRADAAALEHHHHHH*

PelB Leader VH Nluc Nluc VL His Tag

Nluc activity of eluted Alem GloBody during purification using HisPur Cobalt Spin Column after large scale protein expression. Samples diluted 1/1000 in PBS and 25 µL assayed.


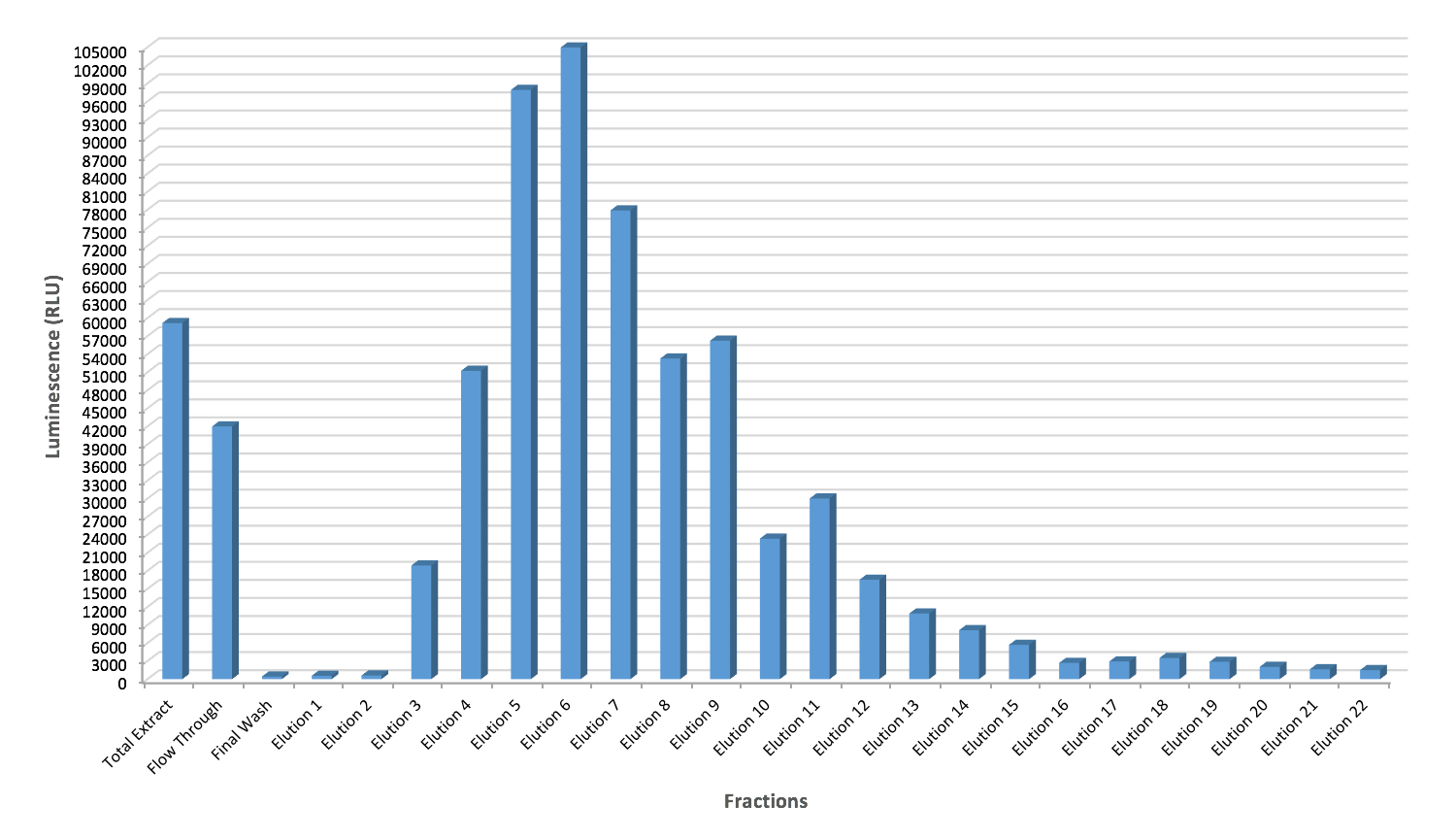


Alem GloBody and Uste GloBody were serially 2 fold diluted from stock and applied to fixed CHO-CD52 cells (96 well plate) in PBS with 10% goat serum. After incubation for 1hr at ambient temperature the wells were washed 3x with PBS and the Glo substrate added and the luminescence determined. The Alem Glo Body titrated out exhibiting maximum binding at 1 (1:2) and reached base line after 11 (1:1024) dilutions. The Ustekinumab based GloBody did not bind. This is indirect evidence that Alem GloBody retains specificity for CD52, the same target as alemtuzumab.

Small Scale Production:

Single colony NEB® Express Iq transformed with pK AlemDnLuc added to 5ml autoinduction media with 100ug/ml kanamycin, grow with shaking at 275rpm for 25hr. Cells pelleted at 3000 rpm for 15 min 0.5ml of the clear supernatant (SUP)retained. The pellet lysed with the addition of 567ul of NPI-10 and 63ul of BugBuster (10x) and 15 units of Benzonase on ice for 30 min and then centrifuged at 12000g for 30 min at 4°C. 20ul of Soluble Extract (SE) retained for SDS PAGE. 600ul loaded onto Ni2+NTA Spin Colum (Qiagen) and centrifuged 1600rpm for 5min and the flow through (FT) retained. Washed with NPI-20 600ul at 3000rpm for 2 min and 1st wash discarded, repeated wash and the final wash (FW) retained. The bound proteins eluted with 2 x 300ul of NPI-500 added for 3min then centrifuged at 3000rpm for 2 min (E1) and (E2).

LDS 4x loading gel with 10% β-mecaptoethanol 15ul, was added to 30ul of each samples (SE, FT, FW and E1), heated to 98°C for 10 min and 25ul run on a 4-12% gradient gel in MES buffer at 200v for 60 min. PageRuler prestained marker was include. The resolved proteins were transferred to a PVDF membrane and blocked with 5% BSA in PBS for 1hr at ambient temperature and the probed with a goat anti-6xHis tag antibody conjugated to HRP (diluted 1:25,000 for 1hr at ambient temperature, washed 5x with PBS 0.05% Tween-20 for 5min per wash and developed with Pierce ECL kit. The developed film aligned with the membrane to locate the 70 kDa and 50 kDa bands and scanned on Konica Minolta photocopier and the scan sent as a pdf by email. The image was copied and pasted below (actual size) no further manipulation or enhancement has been performed.

E1 FW FT SE M

**70 kDa**

**50 kDa**

**65 kDa**

**40-45 kDa**
